# Value of shared preclinical safety studies – The eTOX database

**DOI:** 10.1016/j.toxrep.2014.12.004

**Published:** 2014-12-18

**Authors:** Katharine Briggs, Chris Barber, Montserrat Cases, Philippe Marc, Thomas Steger-Hartmann

**Affiliations:** a Lhasa Limited, Granary Wharf House, 2 Canal Wharf, Leeds LS11 5PS, United Kingdom; b Research Programme on Biomedical Informatics (GRIB), Hospital del Mar Medical Research Institute (IMIM), Department of Experimental and Health Sciences, Universitat Pompeu Fabra, C/Dr Aiguader 88, E-08003 Barcelona, Spain; c Bayer Pharma AG, Bayer HealthCare, Investigational Toxicology, Müllerstrasse 178, D-13353 Berlin, Germany; d PreClinical Safety, Novartis Institute for Biomedical Research, Klybeckstrasse 141, CH-4057 Basel, Switzerland

**Keywords:** ALP, alkaline phosphatase, ALT, alanine aminotransferase, AST, aspartate aminotransferase, CDISC, Clinical Data Interchange Standards Consortium, CRO, contract research organisation, DILI, drug induced liver injury, EFPIA, European Federation of Pharmaceutical Industries and Associations, eTOX, integrating bioinformatics and chemoinformatics approaches for the development of expert systems allowing the *in silico* prediction of toxicities, FN, false negative, FP, false positive, GLP, good laboratory practice, ICH, International Conference on Harmonisation, IMI, Innovative Medicines Initiative, INHAND, International Harmonization of Nomenclature and Diagnostic Criteria, IT, information technology, MCC, Matthews correlation coefficient, OECD, Organisation for Economic Co-operation and Development, PDF, Portable Document Format, PDF/A, ISO-standardized version of PDF specialized for the digital preservation of electronic documents., QA, quality assurance, SEND, Standard for Exchange of Nonclinical Data, SME, small-to-medium enterprise, TN, true negative, TP, true positive, ULN, upper limit of normal, Data sharing, Toxicology, Data mining, Biomarkers, Ontology

## Abstract

A first analysis of a database of shared preclinical safety data for 1214 small molecule drugs and drug candidates extracted from 3970 reports donated by thirteen pharmaceutical companies for the eTOX project (www.etoxproject.eu) is presented. Species, duration of exposure and administration route data were analysed to assess if large enough subsets of homogenous data are available for building *in silico* predictive models. Prevalence of treatment related effects for the different types of findings recorded were analysed. The eTOX ontology was used to determine the most common treatment-related clinical chemistry and histopathology findings reported in the database. The data were then mined to evaluate sensitivity of established *in vivo* biomarkers for liver toxicity risk assessment. The value of the database to inform other drug development projects during early drug development is illustrated by a case study.

## Highlights

• First analysis of the eTOX database for 1214 drugs or drug candidates.
• Shared data mainly from short term <20 days preclinical studies in rat *via* oral route.
• Identified the most frequent treatment related findings.
• Evaluated predictivity of clinical chemistry biomarkers.
• Present a first use case of the database during early drug development.

## Graphical abstract

**Figure:**
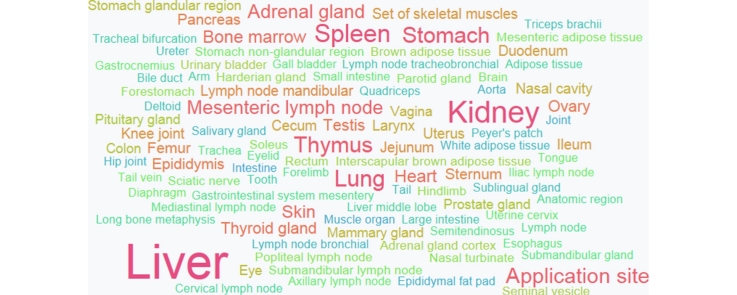

## 1 Introduction

Numerous chemicals are tested by the pharmaceutical industry in order to perform a pre-clinical assessment of clinical safety during the drug development process. Many of these compounds fail during the early preclinical phase and never make it into clinical trials or reach the market [1], [2]. Only rarely are the results of these preclinical studies published. Even if the compound failed, there may be ongoing interest in related compounds which would preclude revealing the chemistry to potential competitors. There may also be a lack of public interest in the failed compound or a lack of enthusiasm from journals for publishing routine toxicology reports. On the other hand, these studies represent a valuable data source for comparison with untested drug candidates or impurities occurring during manufacturing,. It could also help to develop *in silico* predictive models for endpoints which were previously not amenable due to the scarcity of data. In addition, although archived in a fully traceable manner, study reports are rarely stored in a format that supports data mining or the generation of simple statistics. Some pharmaceutical companies have realised this hidden wealth in their archives and started internal work to improve retrievability of their report data. However, such initiatives have remained isolated and often lacked comprehensive data curation steps.

It would clearly be of benefit to the industry to analyse these data across multiple companies in order to learn how to avoid costly failures. By enhancing data availability for compound comparison along with data mining to build more reliable *in silico* predictive models, these data could potentially lead to a more efficient process for drug development and, of broader interest, a reduction in animal use (3Rs principle). However, extracting these data from the reports and building just such a database requires considerable investment in terms of time and money.

In recognition of this, the European Innovative Medicines Initiative (IMI), a public-private partnership of the European Union and the European Federation of Pharmaceutical Industries and Associations (EFPIA), launched a call for a project to be funded to achieve this goal of data sharing and building new *in silico* predictive models. IMI supports collaborative research projects and builds networks of industrial, small-to-medium enterprises (SMEs) and academic experts in order to boost pharmaceutical innovation in Europe. Eleven expressions of interest from consortia of academic institutions and SMEs were submitted for the above-mentioned topic and subsequently evaluated by independent experts during 2008. The project selected was titled “Integrating bioinformatics and chemoinformatics approaches for the development of expert systems allowing the *in silico* prediction of toxicities” and carries the acronym eTOX for electronic toxicity [3]. The eTOX project began January 2010 and as a result of being awarded additional top-up funding is now scheduled to complete its IMI funding phase in December 2016. Total funding over the seven years will amount to 18.7 M€. Table 1 provides a full list of participants.

**Table 1:** Full list of participants in the eTOX project.

Private partners	Public partners
EFPIA companies	Academic institutions
AstraZeneca	Erasmus Universitair Medisch Centrum^a^
**Bayer HealthCare**	European Molecular Biology Laboratory
Boehringer Ingelheim	Fraunhofer Gesellschaft^a^
F. Hoffmann-La Roche	**Fundació Institut Mar d’ Investigacions Mèdiques**
GlaxoSmithKline	Fundación Centro Nacional de Investigaciones Oncológicas Carlos III
H. Lundbeck	Liverpool John Moores University
Janssen Pharmaceutical	Technical University of Denmark
Laboratorios del DrEsteve	Universitat Politècnica de Valencia^a^
Les Laboratoires Servier^a^	Universität Wien
**Novartis Pharma**	University of Leicester^a^
Pfizer Ltd.	Vrije Universiteit Amsterdam
Sanofi^a^	SMEs
UCB Pharma	Chemotargets SL
	Inte:Ligand GmbH
	Lead Molecular Design SL
	Lhasa Limited
	Molecular Networks GmbH
	SYNAPSE Research Management Partners, SL^a^

Organisations leading the project are depicted in bold.

a Organisations that joined eTOX after its inception.

**Fig. 1:**
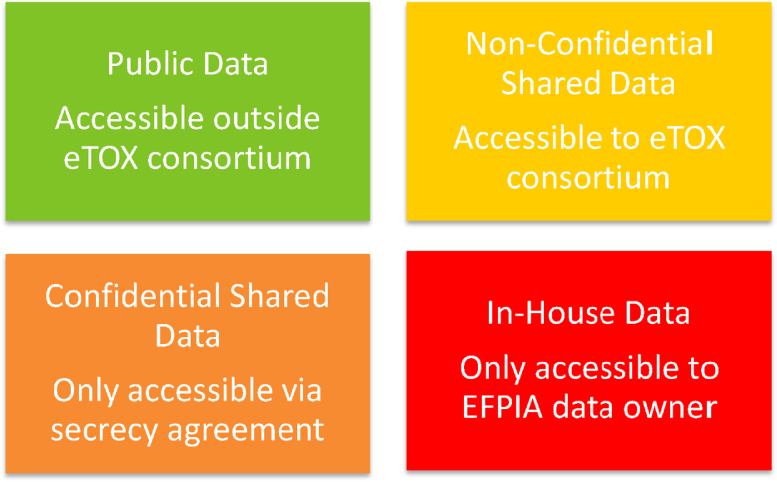
Data sensitivity classifications within the eTOX project.

One key element of the eTOX project is the safe storage of the data extracted from legacy reports generated by the EFPIA partners. Since the beginning of the project it was identified that some data should and will remain confidential at least among the partners in the project; data safety did not only have to cover IT aspects but also safeguarding confidentiality and intellectual property protection. Lhasa Limited, a not-for-profit, charitable organisation with a long history of sharing both confidential and non-confidential data [4], was selected to be the “honest broker” hosting the harmonised toxicity database containing both legacy data donated by the participating pharmaceutical organisations and complementary data collected from the public domain. A combination of legal contracts, physical access controls, software controls and introduction of sensitivity levels (see Fig. 1) were defined in order to safeguard sensitive data shared within the project. Data classified as **Non-Confidential Shared Data** are accessible to all eTOX project participants but are not at this stage available for sharing outside the project. **Public Data** have also been gathered for the purposes of the project and these data are accessible outside the consortium. Data classified as **Confidential Shared Data** are only accessible to the data owner and the honest broker. Model developer partners within the eTOX consortium have to agree a secrecy agreement with the data owner in order to mine these data. **In-house Data** are data that are only accessible by the data owner although not shared with the consortium it is recognised as a category as it could be used to validate eTOX models.

Relevance of the covered chemical space and data quality are essential if the goal of accurate *in silico* toxicity prediction is to be achieved. There are a number of initiatives that are sharing data for toxicity prediction purposes, for example ToxCast^2^ and COSMOS,^3^ but these are not focussed on drug-like chemical space. The legacy data donated includes studies on compounds that have become drugs and many more chemicals that have failed to reach the market whether for safety issues or for other reasons. They are therefore, drug-like and so represent the best possible chemical space for predicting toxicity of future drugs. In addition, the majority of studies will have been conducted in accordance with International guidelines, such as OECD or ICH, and many of them also adhering to the principles of good laboratory practice (GLP). As a consequence the studies can be considered to be of high quality [5].

After setting up the legal and IT infrastructure, EFPIA companies began to identify the study reports which should be extracted and included in the database. Although the scope of the eTOX project is to ultimately cover all *in vivo* study types conducted during drug development, the initial focus was put on routine toxicity studies such as the dose range finding studies, maximum tolerated dose studies, the pivotal studies (mainly 28-day toxicity studies) conducted to support first time in man (FTIM) and the chronic studies aimed at supporting later stage clinical development. The reason for this prioritisation was because they represent the key studies for dose setting and safety assessment prior to FTIM and because it was expected that these study types would occur in the archives with highest frequencies.

**Fig. 2:**
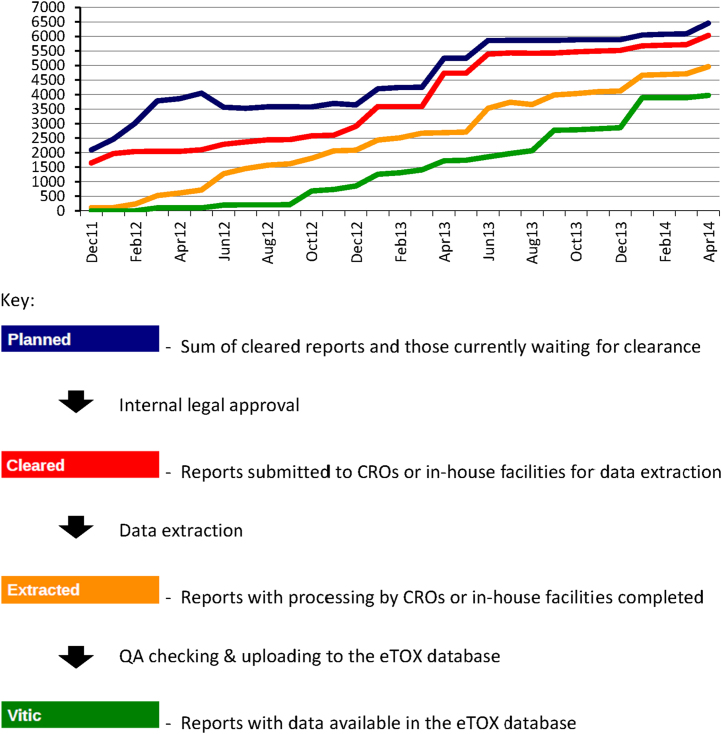
Chronological progress of number and status of study reports as of April 2014.

EFPIA companies found that the majority of these studies were archived as paper files (paper, scanned PDF or PDF/A). After internal legal clearance the manual data extraction was started. Fig. 2 displays the rates at which the study reports were identified, cleared, extracted and included into the Vitic Nexus database management system which is maintained by Lhasa Limited [6].

The eTOX database contains entries for all endpoints measured or determined during a systemic toxicity study. Data are captured for each dose and sex group evaluated in the study plus the control group and all time points where measurements or observations were taken, whether they show an increase, decrease or are unchanged.

For qualitative data such as clinical signs, gross necropsy and histopathology the incidence in each dose group, that is the number of animals recorded as displaying a particular effect and the total number of animals examined for this effect, as well as the severity or grade is captured. For quantitative data such as clinical chemistry, haematology, haemostasis, urinalysis and organ weights the average value for each dose group, standard deviation and units are captured along with the average fold change where this is reported and whether the value was identified as increased or decreased. For both qualitative and quantitative data if the study report records a judgement call on whether the finding is considered treatment-related or not, then this is also captured in the database along with the finding.

A single compound in the eTOX database can have many studies associated with it. These studies are likely to be performed in different species and strains, for different lengths of time with sometimes the compound administered by different routes. This presents a challenge when using the data to build *in silico* models since these factors can all influence the toxicity observed. In addition, each study will have multiple values for a specific endpoint representing each time point at which the data were recorded during the study and the different dose and sex groups. For the purposes of modelling, this information is best condensed down to a single value per chemical, combining results from all studies, dose groups and time points for that chemical. The judgement calls included in the database are useful in this respect as they can be used to derive a binary classification where positive findings are those findings identified in the original study report as treatment-related and negative findings are all findings not identified as treatment-related.

Paramount for the usability of the database was the establishment of ontologies [7] for the numerous terms used throughout the reports. Many of these terms have evolved over time, across different companies, and in the public literature. Often the EFPIA companies did not apply consistent terms in their reports. Therefore the decision was taken to extract the verbatim terms and to then map them retrospectively to a common ontology being developed within the project by a curation team. The hierarchical structure of the ontology also allows findings to be summarised at different levels allowing data captured at different levels of granularity (*e.g.* “gastrointestinal tract” *versus* “colon”) to be used together.

In total, the 13 participating EFPIA pharmaceutical companies have already donated data extracted from 3970 reports to the project and the latest release includes 1214 drugs or drug candidates (Table 2). The eTOX non-confidential shared database is already used within several companies to compare new drug candidates to previously tested compounds [8]. In addition to such comparisons, the database should now have achieved a size to support deeper analyses of potential underlying correlations and for modelling. As a first step, we therefore performed a systematic analysis of distribution of various parameters which are reported in this manuscript.

**Table 2:** Number of data records included in the 2014-1 eTOX non-confidential shared database. Data for compounds that are confidential where the study data are classified as non-confidential are included but without any structural information.

Confidential compounds	Non-confidential compounds	Non-confidential studies
477	737	3393

## 2 Method

The data analysis that follows is based on the 2014-1 version of the eTOX non-confidential shared database released on the 8th April 2014. This incorporates only the non-confidential shared data. If the compound is considered confidential but the study data are classified as non-confidential these data are also included in the release but without any structural information.

**Fig. 3:**
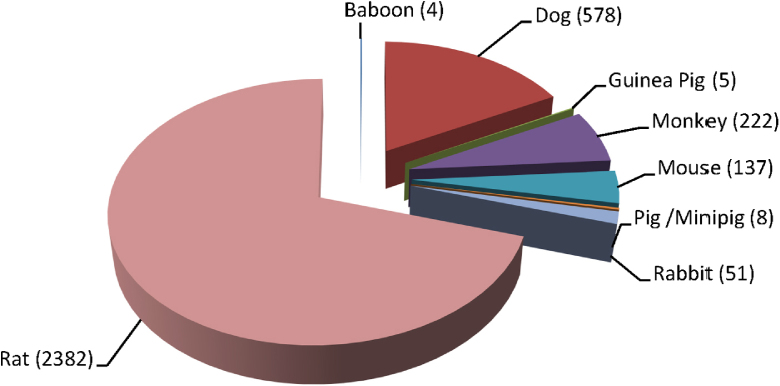
Species distribution in eTOX 2014-1 database (number of studies).

**Fig. 4:**
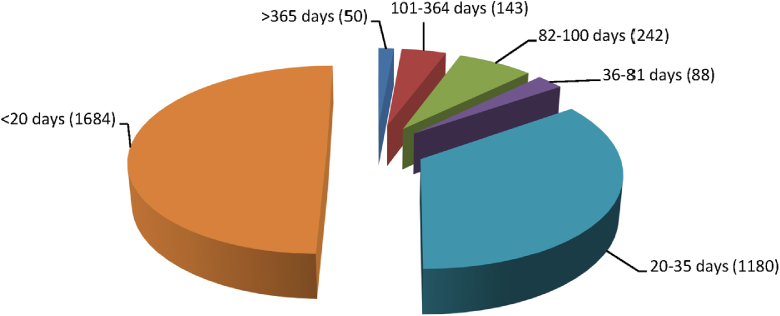
Study duration distribution in eTOX 2014-1 database (number of studies).

**Fig. 5:**
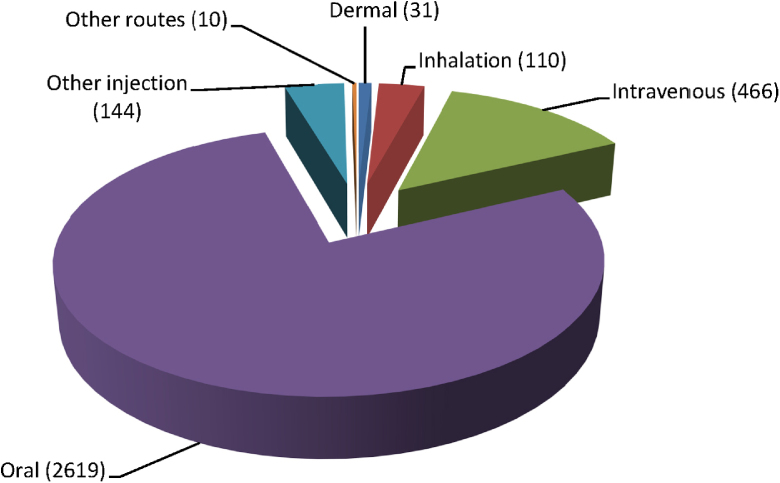
Administration route distribution in eTOX 2014-1 database (number of studies).

KNIME 2.8.0 and Microsoft Excel 2010 were used to perform the data analysis. Database Reader and GroupBy nodes were used to determine how the eTOX non-confidential shared database was split in terms of species, (Fig. 3), duration of exposure (Fig. 4) and administration route (Fig. 5). To create the pie charts, preferred terms that were assigned in the ontology for species and route were used; exposure duration values were assigned to discrete intervals (bins). As the toxicity observed will be affected by these parameters it is important that large enough subsets of homogenous data are available for building models.

Even though many of the extracted reports reflect summarised studies performed according to guidance documents or standard operation procedures, it became clear, that the heterogeneity of terms used for organs, tissues and findings was extremely high. This heterogeneity together with the resulting high numbers of entries describing identical concepts clearly limits the value of the database in terms of query function, statistical analysis and inter-operability. It was therefore key to develop ontologies for mapping of these terms to a smaller number of preferred terms. The consortium ontologies were based on existing ones, such as the Adult Mouse Anatomy Ontology,^4^ and are being developed with other initiatives in mind, such as CDISC-SEND^5^ and INHAND [9]. Many cross references were introduced to maximise interoperability.

**Fig. 6:**
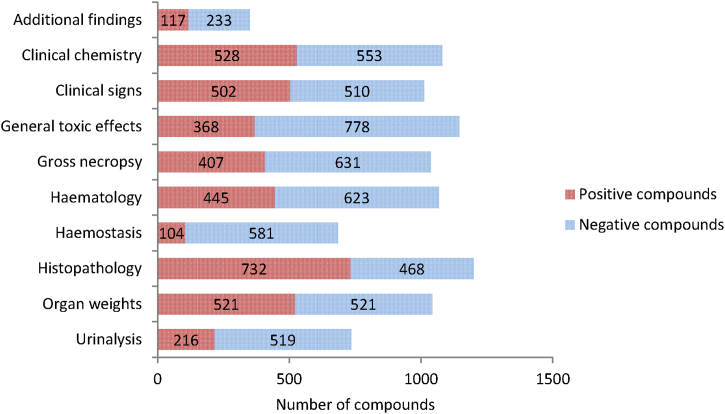
Number of positive *versus* negative compounds associated with the different types of findings in the eTOX 2014-1 database. Positive compounds: compounds with these findings flagged as treatment related. Negative compounds: compounds with these findings that are not flagged as treatment related.

With findings in the database mapped to preferred terms it was possible to analyse the eTOX non-confidential shared data for the most frequent positive findings by counting the number of compounds which had these findings flagged as treatment-related in the study report (Fig. 6). We also investigated the number of negative findings by counting the number of compounds where these findings were not flagged as treatment-related in the study report; as for modelling purposes both negative and positive findings are needed. It is important that those areas with the majority of positive findings are mapped to the ontologies in order to allow grouping of findings at the preferred term level.

**Fig. 7:**
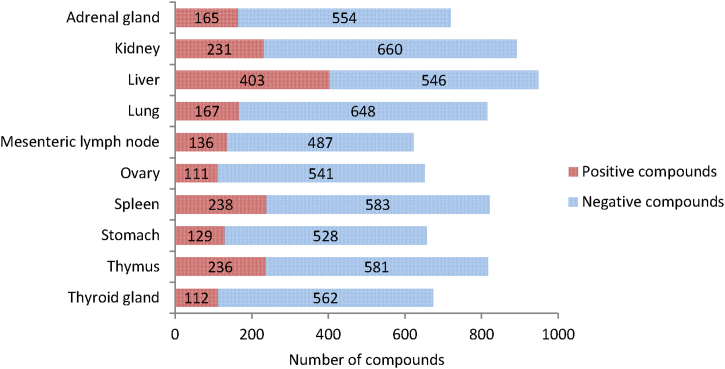
Top 10 organs based on the number of compounds with treatment-related histopathology findings in the eTOX 2014-1 database. Positive compounds: compounds with these findings flagged as treatment related. Negative compounds: compounds with these findings that are not flagged as treatment related.

**Fig. 9:**
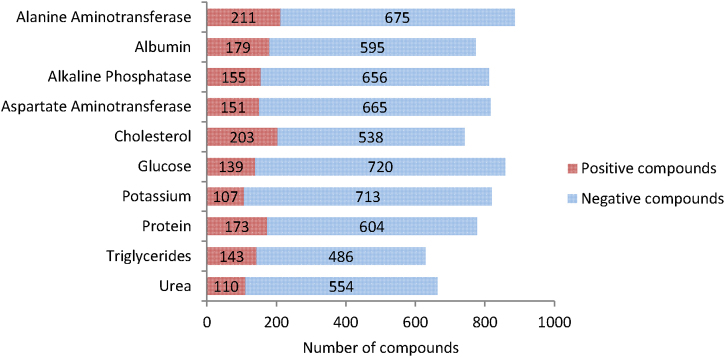
Top 10 changes in clinical chemistry based on the number of compounds with these findings flagged as treatment-related in the eTOX 2014-1 database. Positive compounds: compounds with these findings flagged as treatment related. Negative compounds: compounds with these findings that are not flagged as treatment related.

The majority of verbatim terms for organs and clinical chemistry parameters have already been mapped to these preferred terms in the eTOX ontology (coverage for 2014-1 release at 98% and 80%, respectively). Therefore, we investigated the frequency of organs associated to treatment-related histopathology (Fig. 7) and treatment-related changes in clinical chemistry parameters (Fig. 9).

**Fig. 8:**
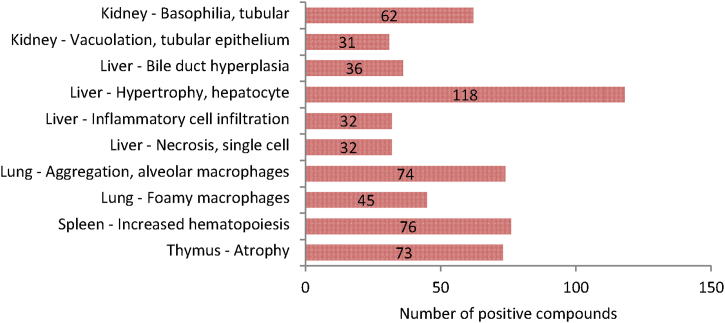
Top 10 histopathology findings based on the number of compounds with these findings flagged as treatment-related in the eTOX 2014-1 database.

Since mapping of histopathology terms to the common ontology is progressing but as yet incomplete (coverage for 2014-1 release at 67%), unmapped terms pending curation were not taken into account for the analysis of frequency of treatment-related histopathology (Fig. 8).

Unsurprisingly, liver was identified as the most common histopathology finding in the database, so we decided to investigate how predictive the different clinical chemistry parameters were of treatment-related histopathology in the liver. Treatment-related refers to the expert call captured from the original report and not one generated by a retrospective analysis of the raw data. See Table 3 for definitions used for this analysis.

**Table 3:** Definitions used for true positive, false positive, false negative and true negative.

	Liver histopathology exists and is treatment-related	Liver histopathology exists and is not treatment-related
Clinical chemistry parameter exists and is treatment-related	True positive (TP)	False positive (FP)
Clinical chemistry parameter exists and is not treatment-related	False negative (FN)	True negative (TN)

Having determined TN, FN, TP and FP values we then used these to calculate Matthews correlation coefficient (MCC) and statistical significance (two tailed Fisher exact test). The clinical chemistry parameters with a significance of p < 0.001 were then ranked using MCC (Table 5).

Rules of thumb to assess human drug induced liver injury (DILI) such as Hy's Law [10] utilise a combination of changes, specifically a 3 fold rise in alanine aminotransferase (ALT) or aspartate aminotransferase (AST) above the upper limit of normal (ULN) accompanied by total bilirubin 2xULN. Alkaline phosphatase (ALP) must also be normal which would exclude cholestatic effects where both bilirubin and ALP are expected to increase. Therefore we also investigated whether predictivity was improved by combining the most predictive of the parameters from the previous analysis, with the other top 10 changes in clinical chemistry. Obviously if both parameters were negative this should result in a combined negative and if both were positive this should be a combined positive but where there was disagreement we had a choice of emphasising either negative or positive predictions. We decided to explore both options for the effects on number of TN, FN, TP and FP as well as MCC values (Table 6).

## 3 Results and discussion

The eTOX 2014-1 database was analysed for distribution by species, duration of exposure and administration route and by frequency of different types of treatment-related findings. The eTOX ontology was used to determine the most common treatment-related clinical chemistry and histopathology findings reported in the database. The data were then mined to evaluate sensitivity of established *in vivo* biomarkers for liver toxicity. The value of the database for the mechanistic assessment of toxic effects of early drug candidates is illustrated by a case study.

### 3.1 Analysis by species

Analysis of how the eTOX non-confidential shared database is split by species reveals that the majority of the data are extracted from rat studies, but that other species including dog and monkey are also well represented.

### 3.2 Analysis by study duration

The split by study duration illustrates that a wide variety of studies are included in the eTOX non-confidential shared database but that donations have been focused on shorter studies of up to 4 weeks in duration.

Besides study duration we also analysed percentage breakdown for each of the different study types in terms of study quality assessment. The majority of the short term studies (<20 days) are described as non-GLP, GLP in part or have no information on study quality (66%), whereas the subacute studies (20–35 days) are predominantly GLP (77%).

### 3.3 Analysis by administration route

In terms of administration route, the vast majority of studies were performed *via* the oral route (gavage or addition to food or drinking water), which reflects the intended dosage form for humans. However, a considerable variety of different routes are represented in the eTOX non-confidential shared database with intravenous being the next most common route.

### 3.4 Frequency of different types of treatment-related findings

Due to the heterogeneity of report protocols, not all finding types are investigated as part of this study. The most prevalent analysis is histopathology, of the 1214 compounds included in the 2014-1 release, 1200 (99%) have histopathology data. The analysis of the frequency of overall treatment-related findings shows that the majority of these are reported under histopathology with 732/1200 (61%) compounds flagged as positive. This is followed by clinical chemistry with 528/1081 (49%) positive compounds although organ weights and clinical signs are of a similar magnitude. In terms of the ratio of positive *versus* negative compounds; histopathology has overall more positive findings whereas clinical chemistry has roughly equal numbers of positive and negative findings.

### 3.5 Frequency of treatment-related histopathology findings

Investigating the histopathology findings further reveals that out of the 229 distinct locations included in the eTOX non-confidential shared database, liver is the most common organ to show drug-related effects with 403 positive compounds. This is followed by spleen as the second most common organ with 238 positive compounds although kidney and thymus are of a similar magnitude. The list of organs investigated per study vary, for example of the 1200 compounds having histopathological findings associated with them, only 949 (79%) have data for the liver.

We hypothesised that the high ranking of some of these organs, *e.g.* thymus, could be the consequence of toxicity in other organs where the first organ is the primary target and the thymus a secondary target [11]. In order to assess this, we removed findings where the lowest dose associated with a treatment-related finding in the thymus was higher than the overall lowest dose for all histopathology findings in the study. Using this method, the number of positive compounds drops from 236 to 136. Looking at which organs are associated most frequently with treatment-related findings in the thymus then of these 136 substances 74 also showed effects in the liver and 73 in spleen.

We also considered the effects on this ranking due to the distribution of study types within the database. Since almost half of the studies are of short duration (<20 days) this might skew the results to organs more exposed to the drug or more susceptible to toxicity in the short term and underestimate organs where pathology develops over longer periods of time. We therefore reanalysed the data for each study type to see whether there were any differences. Liver remained as the number one finding for all study types and both spleen and kidney remained ranked in the top 10 (Table 4).

**Table 4:** Top 10 organs for each study type based on the number of compounds with treatment-related histopathology findings in the eTOX 2014-1 database.

Rank	<20 days	20–35 days	36–81 days	82–10 days	>101–364 days	>365 days
1	Liver	Liver	Liver	Liver	Liver	Liver
2	Thymus	Kidney	Thymus	Adrenal gland	Kidney	Spleen
3	Spleen	Spleen	Lung	Lung	Adrenal gland	Testis
4	Kidney	Thymus	Mesenteric lymph node	Spleen	Thymus	Gall bladder
5	Lung	Lung	Spleen	Thymus	Lung	Kidney
6	Stomach	Adrenal gland	Bone Marrow	Testis	Testis	Ovary
7	Adrenal gland	Mesenteric lymph node	Epididymis	Kidney	Spleen	Mammary gland
8	Mesenteric lymph node	Ovary	Kidney	Stomach	Mesenteric lymph node	Adrenal gland
9	Duodenum	Stomach	Ovary	Skin	Ovary	Brain
10	Heart	Thyroid gland	Vagina	Thyroid gland	Stomach	Heart

In the case of pathology terms mapped to the eTOX ontology the top 10 findings observed are located in 5 organs: kidney, liver, lung, spleen and thymus, all of which were among the top 10 organs identified in the previous analysis. Unsurprisingly, hepatocyte hypertrophy is the top finding reported followed by increased hematopoiesis in the spleen.

However since the verbatim pathology terms have not been fully mapped to the ontology (coverage currently at 67%) these should be considered preliminary findings. When dealing with data from 13 companies, produced by hundreds of pathologist, a great deal of normalisation is needed in order to be able to do cross study analysis efficiently. It is a work in progress with a core team of pathologists from four distinct companies meeting every two weeks along with connections to controlled vocabularies (standards like INHAND and SEND terminology initiatives). As of 13th March 2014 the histopathology finding ontology contained 848 preferred terms and 18,808 synonyms.

### 3.6 Frequency of treatment-related clinical chemistry findings

On analysing clinical chemistry the most prevalent finding here is treatment-related changes in ALT with 211 positive compounds, confirming the liver as a top contributor to compound toxicities. Effects on cholesterol, albumin and protein are also common. AST and ALP also make the top 10 but not bilirubin. Considering the preponderance of histopathology effects in the liver it is perhaps not surprising that, with the exception of potassium, these are all indicators of hepatic injury [12]. Treatment-related changes in potassium could be due to gastrointestinal disorders or damage to the kidney [13].

### 3.7 Biomarkers for liver toxicity

Bilirubin does not make the top 10 most common treatment-related clinical chemistry findings in the database, however, it does make the top 10 for clinical chemistry changes predictive of treatment-related histopathology findings in the liver. The table below reports the values for true and false positives and negatives for the top 10 statistically significant parameters ranked by MCC value (Table 5).

**Table 5:** Top 10 clinical chemistry changes predicting histopathology in the liver.

Clinical chemistry parameter	TP	FP	FN	TN	MCC	Sensitivity	Specificity
Alanine aminotransferase (ALT)	135	64	206	392	0.2921	39.6	86.0
Alkaline phosphatase (ALP)	106	43	217	371	0.2771	32.8	89.6
Cholesterol	124	61	174	303	0.2755	41.6	83.2
Aspartate aminotransferase (AST)	93	49	226	374	0.2211	29.2	88.4
Bilirubin	53	27	198	322	0.1941	21.1	92.3
Triglycerides	84	54	172	261	0.1820	32.8	82.9
Albumin	99	61	223	306	0.1669	30.7	83.4
Creatinine	59	33	247	374	0.1650	19.3	91.9
Urea	61	43	199	301	0.1438	23.5	87.5
Protein	92	66	226	311	0.1358	28.9	82.5

Combining ALT, the most predictive of these parameters, with the other clinical chemistry parameters included in the top 10, so that if one is positive and the other negative this is treated as a combined negative, increased the number of true and false negatives and reduced true and false positives. However in terms of MCC values none of the combined parameters performed better than ALT alone.

By contrast higher MCC values were obtained if ALT was combined with other parameters so that if one is positive and the other negative it is considered a combined positive (Table 6). The increase in MCC was accompanied by increased true positives and decreased false negatives. False positives were also decreased when combining ALT with ALP, AST, bilirubin, creatinine and urea. However, only the combination of ALT and bilirubin increased true negatives.

**Table 6:** Effect of combining clinical chemistry changes on predicting histopathology in the liver.

Clinical chemistry parameter	FP	TP	TN	FN	MCC	Sensitivity	Specificity
ALT + alkaline phosphatase	53	168	379	180	0.3972	48.3	87.7
ALT + creatinine	44	157	384	192	0.3942	45.0	89.7
ALT + cholesterol	74	182	378	168	0.3790	52.0	83.6
ALT + bilirubin	47	150	393	196	0.3742	43.4	89.3
ALT + aspartate aminotransferase	54	149	389	194	0.3541	43.4	87.8
ALT + triglycerides	72	168	377	180	0.3486	48.3	84.0
ALT + albumin	70	166	379	183	0.3475	47.6	84.4
ALT + urea	59	155	374	192	0.3457	44.7	86.4
ALT + protein	72	167	373	182	0.3427	47.9	83.8
ALT	64	135	392	206	0.2921	39.6	86.0

### 3.8 Case study for evaluation and assessment of toxic effects of early drug candidates

Besides data mining the database for the sensitivity of established *in vivo* biomarkers as described above it can also have a value for the mechanistic assessment of toxic effects of early drug candidates This shall be illustrated by the following example.

**Fig. 10:**
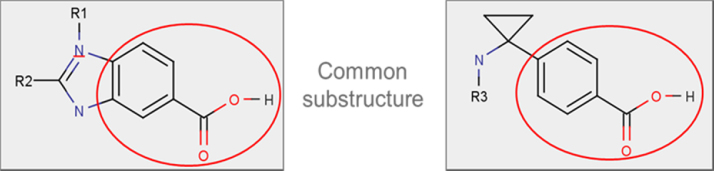
Investigated compounds for which similar haematological findings were observed.

For the investigation of potential drug candidates for a target related to the prostaglandin E receptor, two compounds were investigated in a short term rat toxicity study (see Fig. 10).

Both compounds resulted in the following haematological findings:

• decrease in erythrocytes and haemoglobin
• increase in reticulocytes
• increase in thrombocytes
• increase in leucocytes and neutrophils

Since both compounds contain a benzoic acid moiety, medicinal chemists asked if these rather specific haematological findings could be related to the substructure and whether removal of this substructure could alleviate these findings, prompting a search of the eTOX database for evidence to support this hypothesis.

In a first step the prevalence of the benzoic acid moiety was determined in the whole dataset. In a subsequent query the number of compounds containing the benzoic acid moiety and concomitantly causing any kind of haematological findings was determined.

**Fig. 11:**
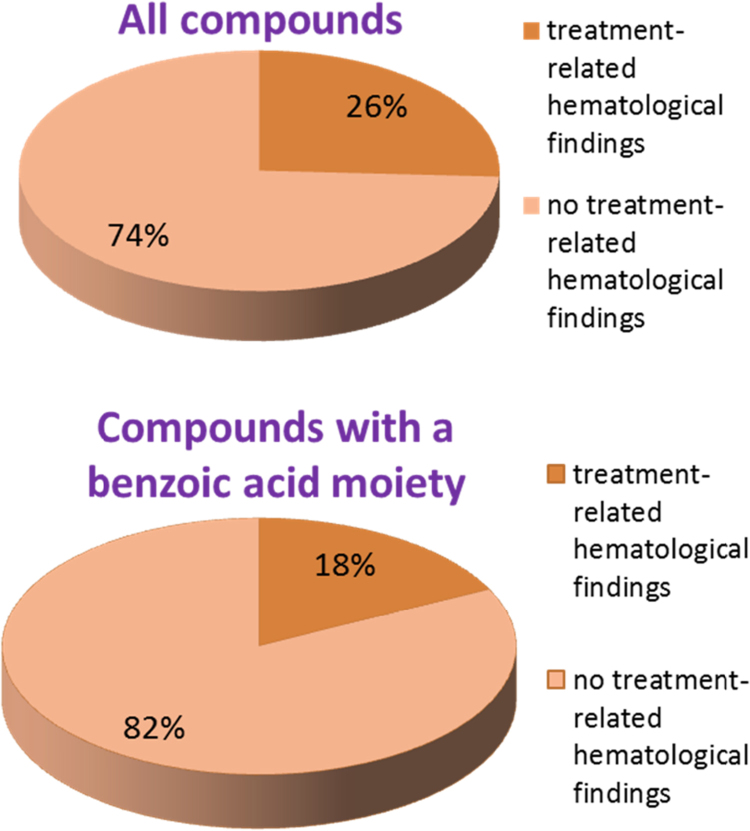
Percentage of compounds associated with haematological findings. Upper pie chart: for all compounds in the eTOX 2014-1 database. Lower pie chart: the 1.1% of compounds containing a benzoic acid moiety.

The results of these queries are displayed in Fig. 11.

Overall the free benzoic acid moiety occurred in only 1.1% of all queried compounds. Only 18% of the compounds with this structural feature were associated with the specified treatment-related haematological findings, while 26% of all queried compounds had treatment-related haematological findings. It is therefore concluded that there is no evidence that the benzoic acid moiety is over-represented in the group of compounds with treatment-related haematological findings, which speaks against a causal relationship of this substructure.

**Fig. 12:**
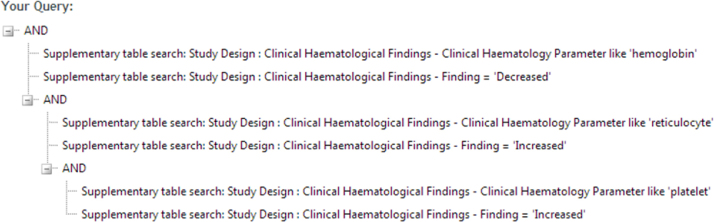
Screenshot of the multi-parameter search for findings observed in the short term toxicity studies with the relevant drug candidates.

In a second step the database was queried for the occurrence of the combined findings; decrease in haemoglobin and concomitant increase in both platelets and reticulocytes (Fig. 12).

The query resulted in a list of 20 compounds. Upon inspection of these 20 hits, two compounds attracted specific attention (see Table 7) not only because they showed a structural similarity to the compounds under question, though the benzene ring was replaced by a pyrazin, but also because the information on the pharmacological action of the compound pointed to a related target (EP1 receptor antagonist). In addition, the query also revealed that the findings are not restricted to rat studies but were also observed in dogs, *i.e.* the observed effects do not seem to be species-specific and the weight of evidence points towards a pharmacodynamic action rather than a pure chemistry-related toxicity.

**Table 7:** Selected compounds of the multi-parameter search for decrease in haemoglobin and concomitant increase in both platelets and reticulocytes. The two compounds show both a structural similarity with the early candidates depicted in Fig. 10 and a pharmacological mode of action related to the described development project.

Pharmacological action	Structure image	Species	Strain	Sex	Vehicle	Dosage
EP1 receptor antagonist		Rat	Alpk:APfSD Wistar derived	Male and female	0.5% hydroxypropyl methylcellulose in 0.1% aqueous polysorbate 80	0 mg/kg; 5 mg/kg; 50 mg/kg; 2000 mg/kg reduced to 1000 mgkg on day 18

EP1 receptor antagonist		Rat	AP rats (Alpk:APfSD strain, Wistar derived)	Female	0.5% (w/v) HPMC solution containing 0.1% (w/v) polysorbate 80	0 mg/kg/day; 50 mg/kg/day; 300 mg/kg/day; 1200 mg/kg/day

EP1 receptor antagonist		Rat	AP rats (Alpk:APfSD strain, Wistar derived)	Male	0.5% (w/v) HPMC solution containing 0.1% (w/v) polysorbate 80	0 mg/kg/day; 50 mg/kg/day; 300 mg/kg/day; 1000 mg/kg/day

EP1 receptor antagonist		Dog	Beagle	Male and female	0.5% (w/v) hydroxylpropyl methylcellulose (HPMC) solution containing 0.1% (w/v) aqueous polysorbate 80	0 mg/kg; 75 mg/kg; 150 mg/kg; 300 mg/kg

In summary, the database delivered valuable contributions to this development project with regard to chemistry-related toxicity, species-specificity of observed toxic effects and selectivity of the pharmacodynamic action of the compound without any additional animal experiments.

## 4 Conclusion

The eTOX database is potentially the largest available repository of *in vivo* repeat dose toxicity data derived from industry drug discovery activities. Its development means that this vast wealth of legacy data previously only accessible to the respective data owners can now be more fully exploited. By collaboratively sharing these data, it is no longer a seemingly impossible task to answer simple questions such as ‘what is the most common drug-induced liver toxicity across all studies?’; in this present analysis hepatocyte hypertrophy. In the future, many more complex questions will be answered using this valuable toxicological resource. The description of a first use case shows how the database can be meaningfully applied to answer questions raised during early drug development without performing additional animal studies.

The initial focus of data collection by pharmaceutical companies participating in the project has been systemic toxicity, repeat dose studies in preclinical species. However during the course of the project integration with existing clinical databases and extraction of data from safety pharmacology, pharmacokinetics, pharmacodynamics, reproductive-developmental toxicity and carcinogenicity studies is planned, along with use of all these data to build robust multiscale and multilevel predictive models to support risk assessment. The intention is not to create a frozen system with static data and models but to continue data incorporation beyond the end of the project and drive modelling practice. Drug development data are becoming increasingly available to the community and we do believe precompetitive exchanges should become the norm in the future. As part of the discussion on sustainability the consortium is also considering how the database and models integrated into eTOXsys, the predictive system developed within the eTOX consortium, might be made more widely available to the scientific community post project.

## Conflict of interest

C. Barber and K. Briggs report that the work under consideration for publication was funded by the Innovative Medicines Initiative Joint Undertaking under grant agreement number 115002. In addition, Lhasa Limited is a not-for-profit membership organisation whose members, including several EFPIA partners, contribute to the future of Lhasa Limited software and the company.

All authors are members of the IMI-eTOX consortium. Dr. Marc reports that his company (Novartis), via the IMI system contributed to eTOX. Outside of the IMI scope, Novartis is buying licenses for some of the Lhasa limited tools.

Dr. Steger-Hartmann is an employee of Bayer Pharma AG.

## Transparency document

Transparency document

## Footnotes

2 http://www.epa.gov/ncct/toxcast/ (accessed 16.09.14).

3 http://www.cosmostox.eu/home/welcome/ (accessed 16.09.14).

4 http://www.obofoundry.org/cgi-bin/detail.cgi?id=adult_mouse_anatomy (accessed 16.09.14).

5 http://evs.nci.nih.gov/ftp1/CDISC/SEND/SEND%20Terminology.html#CL.C650 (accessed 16.09.14).
